# From Isolated Emotional Memories to Their Competition During Conflict

**DOI:** 10.3389/fnbeh.2020.00036

**Published:** 2020-03-12

**Authors:** Christian Bravo-Rivera, Francisco Sotres-Bayon

**Affiliations:** ^1^Cold Spring Harbor Laboratory, Cold Spring Harbor, NY, United States; ^2^Institute of Cell Physiology—Neuroscience, National Autonomous University of Mexico, Mexico City, Mexico

**Keywords:** aversion, risk, reward, valence, prefrontal cortex, amygdala, nucleus accumbens, avoidance

## Abstract

Aversive or rewarding experiences are remembered better than those of lesser survival significance. These emotional memories, whether negative or positive, leave traces in the brain which can later be retrieved and strongly influence how we perceive, how we form associations with environmental stimuli and, ultimately, guide our decision-making. In this review aticle, we outline what constitutes an emotional memory by focusing on threat- and reward-related memories and describe how they are formed in the brain during learning and reformed during retrieval. Finally, we discuss how the field is moving from understanding emotional memory brain circuits separately, towards studying how these two opposing brain systems interact to guide choices during conflict. Here, we outline two novel tasks in rodents that model opposing binary choices (approach or avoid) guided by competing emotional memories. The prefrontal cortex (PFC) is a major integration hub of emotional information which is also known to be critical for decision-making. Consequently, brain circuits that involve this brain region may be key for understanding how the retrieval of emotional memories flexibly orchestrates adaptive choice behavior. Because several mental disorders (e.g., drug addiction and depression) are characterized by deficits in decision-making in the face of conflicting emotional memories (maladaptively giving more weight to one memory over the other), the development of choice-based animal models for emotional regulation could give rise to new approaches for the treatment of these disorders in humans.

## Introduction

An emotional memory represents the storage of information about a survival experience. Through associative mechanisms, neurons assign emotional significance to environmental stimuli, and these memories influence motivation to behave adaptively. Thus, individuals revisit places previously associated with reward, whereas they avoid ones previously associated with aversion.

Emotional memories can be categorized along two dimensions: salience ranging from weak to strong, and valence ranging from negative (aversive) to positive (rewarding). The salience of an emotional memory represents the apparent impact of the experience, and it correlates with arousal intensity. Emotionally-charged memories are better retrieved than neutral ones (Conway et al., 1994). Indeed, stress responses elicited during salient situations facilitate memory formation (McGaugh, 1983). Importantly, salience signals that something relevant is occurring and it is dissociable from valence (Lin and Nicolelis, 2008). The valence of an emotional memory represents the value of the experience; whether environmental elements were paired with pleasant or aversive experiences. These value- and saliency-based signaling are encoded in neural circuits that involve the amygdala, nucleus accumbens (NAcc) and prefrontal cortex (PFC), which ultimately guide behavior (Rangel et al., 2008; Tye, 2018).

Many groups focus on dissecting the contribution of neural circuits connecting them in valence and salience, partly to understand emotional processing (Namburi et al., 2016). Several decades of research on aversive memories have focused primarily on the contribution of the amygdala in threat (fear) conditioning. Also, decades of research on appetitive and drug-seeking memories have focused primarily on the NAcc in reward encoding. Although there is overlap (Xiu et al., 2014), encoding of aversive memories occurs in several structures including the amygdala, prelimbic (PL) PFC and periaqueductal gray matter (PAG) whereas encoding of reward or safety memories occurs mainly in the NAcc, ventral tegmental area (VTA), as well as infralimbic (IL) prefrontal and orbitofrontal cortices (OFC; Peters et al., 2009; Sotres-Bayon and Quirk, 2010). This is not to say that structures are exclusively dedicated to either reward or aversion processing; the amygdala is also critical for reward processing (Balleine et al., 2003) whereas NAcc mediates aversion as well (Berridge, 2019). The reward and aversion models we describe below are not comprehensive, but rather, simplified models that are suitable as an outline for reward and aversion circuits.

## Aversive Memories

**Figure 1:**
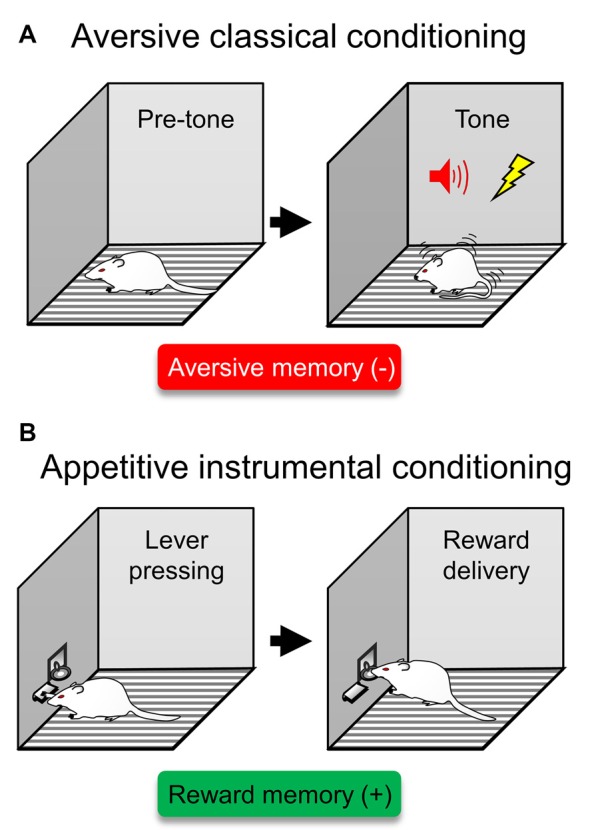
Traditional behavioral assays to probe isolated aversive or reward memories. **(A)** A type of aversive classical conditioning is auditory threat (fear) conditioning. In this type of aversive learning, rodents are presented with a tone that predicts a foot-shock. After several exposures of this tone/shock pairings, rodents express defensive responses in the form of conditioned freezing. This behavioral assay has been traditionally used to probe aversive memories (red box), representing a previous experience that acquired a negative valence (−). **(B)** In appetitive instrumental conditioning, rodents learn to press a lever to obtain reward, often in the form of food. This behavioral assay has been traditionally used to probe reward memories (green box), representing a previous experience that acquired a positive valence (+).

Learning to detect and respond to threats is necessary for survival. Individuals remember aversive situations, such that they subsequently avoid stimuli previously associated with aversion. These aversive experiences are stored as emotional memories that guide our behavior. The most fruitful advances in the neurobiology of aversive encoding have been achieved by studying Pavlovian threat conditioning (Davis, 1992b; LeDoux, 1996). Threat conditioning involves learning that a neutral stimulus, such as a tone, predicts a co-terminating aversive event, such as a shock. Subsequent presentations of the tone evoke defensive behaviors that include freezing and avoidance (Figure 1A).

The neural circuit involving this type of conditioning centers on the amygdala (Davis, 1992a; LeDoux, 1992), a structure with well-suited connections to detect and avoid the threat. Information about the tone and shock converge in the amygdala directly from sensory thalamus (LeDoux et al., 1990; Quirk et al., 1995). Synaptic plasticity mechanisms within the amygdala have been identified as a key component underlying the ability to learn and store this type of emotional experience (Rodrigues et al., 2004). After the initial synaptic strengthening that occurred during learning-related plasticity, other processes take over to maintain the potentiation of synapses, thereby keeping the memory in long-term storage (Sacktor, 2008).

When retrieving the conditioned threat memory, amygdala neurons trigger defensive responses *via* descending projections to midbrain structures, such as the PAG, involved in freezing (LeDoux et al., 1988; Amorapanth et al., 1999), or the NAcc, involved in active threat avoidance (Amorapanth et al., 2000; Bravo-Rivera et al., 2014; Ramirez et al., 2015). Importantly, the amygdala has strong reciprocal connections with PFC (Gabbott et al., 2005), which allows PL to upregulate fear responses generated by the amygdala (Burgos-Robles et al., 2009; Sotres-Bayon et al., 2012). Thus, expressing a fear memory involves the concerted activity of a distributed neural circuit.

Because aversive memories stored in the defensive survival circuit can last a lifetime (Gale et al., 2004), their expression needs to be highly regulated. One way to suppress obsolete fear memories is through fear extinction, in which subjects learn that the once-threatening stimulus no longer predicts danger (Pavlov, 1927; Rescorla, 2001). After threat conditioning, repeated exposure to unreinforced tones progressively results in reduced freezing (Bouton and Bolles, 1980). However, fear is not unlearned or erased; rather, a safety memory is formed that suppresses the original fear memory (Lolordo and Rescorla, 1966; Quirk and Mueller, 2008). Understanding the neural circuit that underlies fear extinction promises to improve methods to treat fear-related disorders (Anderson and Insel, 2006). Because extinction is the basis of exposure-based therapy in humans, its neuronal circuits are currently under intense investigation (Sotres-Bayon et al., 2006; Quirk and Mueller, 2008). This neuronal circuit involves both the amygdala and PFC (Sotres-Bayon et al., 2007, 2009). In brief, the current circuit model for extinction requires that information about the conditioned tone from the amygdala reaches IL. After presentations of tones in the absence of the shock, IL facilitates plasticity in the amygdala that stores the extinction memory (Amano et al., 2010; Amir et al., 2011; Cho et al., 2013; Do-Monte et al., 2015). Plasticity changes in amygdala neurons store the extinction memory and inhibit fear-generating neurons, thereby reducing fear expression. Yet extinction is a passive form of fear suppression, and often the adaptive response needs to be immediate to either actively suppress fear in order to obtain a reward or to actively avoid threatening stimuli or places. Fear memories not only trigger reactive freezing but also trigger deliberate actions in the face of threats such as avoidance. Evidence suggests that the amygdala is necessary for avoidance behaviors (Choi et al., 2010; Bravo-Rivera et al., 2014; Ramirez et al., 2015). Interestingly, regulation of these amygdala-dependent avoidance responses requires PL encoding (Bravo-Rivera et al., 2014, 2015a), which controls behavioral output through projections to NAcc, a limbic-motor interface (Bravo-Rivera et al., 2015b; Floresco, 2015; Diehl et al., 2019).

Retrieval of fear memories involves activation of neuronal circuits containing previously potentiated synapses. When retrieving the memory, synapses undergo re-strengthening. This process is called *reconsolidation* (Alberini and Ledoux, 2013). Because synapses undergo restructuration during reconsolidation, this process renders memories labile to editing; this allows current memories to interact with the retrieved ones. For example, during the reconsolidation of a fear-memory, new information can be integrated, such as if the cue was present in a different context from the original dangerous experience (Nader and Hardt, 2009). The susceptibility of memories during reconsolidation is currently explored as a therapeutic target, such that administering drugs that impair reconsolidation may disrupt the fear memory, thereby alleviating excessive fear in patients (Kindt et al., 2009). The reviewed brain mechanisms that underlie formation/reformation of aversive memories can occur simultaneously with those of memories with the opposite valence, reward, such that memories can have both positive and negative associated valences.

## Reward Memories

Natural rewards produce pleasure and are necessary for species preservation, such as with food and sex. To benefit from the experience, individuals learn to associate stimuli and actions with the availability of rewards. A commonly used assay to study reward learning is appetitive instrumental conditioning (Cardinal et al., 2002), in which an action such as pressing a bar leads to a reward such as food (Figure 1B). The neural circuit that underlies this approach behavior has been widely studied and involves the VTA and NAcc (Parkinson et al., 2000; Martin-Soelch et al., 2007). In a series of experiments in the 1950s, Olds and Milner (1954) found that rats will press a lever to self-stimulate the VTA to NAcc pathway at an even faster rate than to obtain food. Activity in this pathway is responsible for learning to predict future rewards (Schultz and Dickinson, 2000) and storing reward associations (Kalivas and Nakamura, 1999). Thus, synaptic plasticity in this circuit permits associations to be formed between stimuli/responses and rewards. The VTA projects primarily to NAcc/PFC and receives input from the lateral hypothalamus that detects the presence of food reward (Schultz, 1998). The NAcc is the ventral region of the striatum, the main input nucleus of the basal ganglia and the site of action for most addictions (Kalivas et al., 2006). In turn, the NAcc sends axons to brain regions involved in the movement, including the globus pallidus and, *via* the thalamus-PFC relay, motor cortices. In brief, in instrumental conditioning, the response associated with the availability of a reward excites the pathway from the VTA to NAcc, which in turn triggers an approach response by acting on the motor system (Yun et al., 2004; Fields et al., 2007).

Retrieval of the cue associated with the reward triggers the excitation of VTA. In reward conditioning, rodents learn that a sensory stimulus (e.g., light) predicts a reward. When presented with reward, VTA neurons release dopamine in PFC and NAcc, which together with a concurrent surge of norepinephrine, stores the salient reward memory with positive valence. After learning, the reward memory is stored in the PFC and NAcc, and activation of the PFC-NAcc circuit that participated in storing the reward memory results in retrieval of the memory (Peters et al., 2005, 2009; Kalivas et al., 2006).

Vast research resources are directed to understand the underpinnings of reconsolidation and extinction of reward memories partly because tampering with these processes may serve as a therapy for addictions (Otis et al., 2015). Moreover, like fear extinction, drug-seeking extinction is also mediated by PFC. In fact, in drug-seeking as in fear conditioning, the same dorsal-ventral dichotomy (PL-IL) function of PFC is involved, but *via* divergent projections to the NAcc rather than to the amygdala (Peters et al., 2009). Notwithstanding, the approach system must interact and even suppress the aversive system to attain reward. Therefore, to have a complete understanding of the neural circuit that supports approach behaviors, it is necessary to understand how reward interacts with the neural circuit controlling opposing actions such as avoidance behaviors.

## Interaction of Competing Emotional Memories Circuits

A burning question in the field of emotion neurobiology is how does the brain integrates different types of emotional experiences to achieve an adaptive behavioral response? There are several structures that engage in balancing competing drives that have been characterized, to some extent. The resolution of this emotional conflict is thought to be critically mediated by cortical subregions of PFC integrating information from and exerting its influence over downstream subcortical structures. Next, we discuss how the PFC has been involved in processing competing emotional memories.

A characteristic feature of PFC is that it is a massive information hub, receiving input from other cortices, thalamus, amygdala, and the hippocampus, among others, and it is likely involved in deliberate decision-making. The central components of the aversion and reward circuits are highly interconnected with the PFC (Peters et al., 2009). Indeed, damage to this cortical region impairs the ability to flexibly select suitable behaviors (Sotres-Bayon et al., 2006; Schoenbaum et al., 2009). A currently popular notion of PFC suggests its key role in emotional processing and decision making (Damasio, 1996; Miller and Cohen, 2001) which often depends on weighing information about aversion and reward memories to control behavior. The PFC may exert its conflict resolution function through connections with other structures, such as the striatum (Berendse et al., 1992), thalamus (Choi et al., 2019), ventral hippocampus (Schumacher et al., 2018) and amygdala (McDonald et al., 1996; Gabbott et al., 2005), to name a few. A broad range of studies has shown that medial PFC (mPFC) encodes both rewarding (Otis et al., 2015) and aversive stimuli (Burgos-Robles et al., 2009). Enhancing activity in PL increases fear and drug-seeking, while activity in IL decreases fears and drug-seeking, through projections to the amygdala and the NAcc, respectively. These findings suggest that mPFC provides control of the amygdala for aversive memories and of the NAcc for reward memories (Peters et al., 2009). On the other hand, damage to OFC impairs reversal learning of both aversive and appetitive association (Murray et al., 2007). In addition, although most OFC studies have focused on its role in processing reward value, there is also evidence that OFC signals aversive stimuli (Morrison and Salzman, 2011). In fact, a recent report shows that neurons in OFC can respond to both aversive and appetitive stimuli (Morrison et al., 2011) and guide choice-behavior (Ramírez-Lugo et al., 2016). This suggests that OFC weighs information from these opposing stimuli and regulates behavior. Further, the PFC has direct access to/from a set of brain structures that signal context (hippocampus Smith and Bulkin, 2014), “controllability” (raphe nucleus Maier and Watkins, 2005) and perception (sensory thalamus as well as sensory cortices). Taken together, these findings suggest both IL/PL and OFC coordinate the interaction between threat- and reward-related stimuli. The working hypothesis is that PFC is responsible for orchestrating the interaction between the drive to avoid and the drive to approach, thus allowing for flexible emotional regulation (Sotres-Bayon and Quirk, 2010).

## Using Conflict Choice Behavior to Understand Competing Emotional Memories

In our everyday life, we are frequently challenged with emotional conflicts. We are often challenged with opportunities of reward with a risk of aversion, and sometimes reward attainment involves imminent aversion. The field has made great strides in characterizing neuronal systems of reward-seeking and aversion avoidance, and with the development of new technologies, it is now timely to probe how these two systems compete for control of behavior. Many behavioral tasks that involve evaluating approach and avoidance motivated behaviors have been developed (Elliot, 2008). Yet only recently a few have evaluated them when in competition during conflict. Here, we outline two novel conflict tasks in rodents that model opposing binary choices (approach or avoid) guided by competing emotional memories.

Most previous studies of emotional memory regulation use tasks where individuals have only one behavioral goal; either approach a reward or avoid a threat. In these cases, emotional memories are studied in isolation. But in nature, when an animal is challenged to choose between approach or avoidance drives, both systems interact. To understand the interaction between competing emotional memories, we need to look at the nature of the choice-mediated conflict. This involves considering the weights of each emotional memory because animals choose what behavior to execute guided by the relative weighs of opposing memories. Both early and recent works have made efforts to characterize approach-avoidance conflict (Miller, 1944; Choi and Kim, 2010; Friedman et al., 2015; Burgos-Robles et al., 2017; Schumacher et al., 2018; Choi et al., 2019; Verharen et al., 2019; Walters et al., 2019). We recently developed two related approach-avoidance conflict animal models that are set to separate discrete variables (reward memory retrieval, threat memory retrieval and their competition) in the same individual by putting variable weigh on reward- or threat-related memories through training, and therefore are amenable to study both sides of the coin: when reward has higher relative value than threat and vice versa.

**Figure 2:**
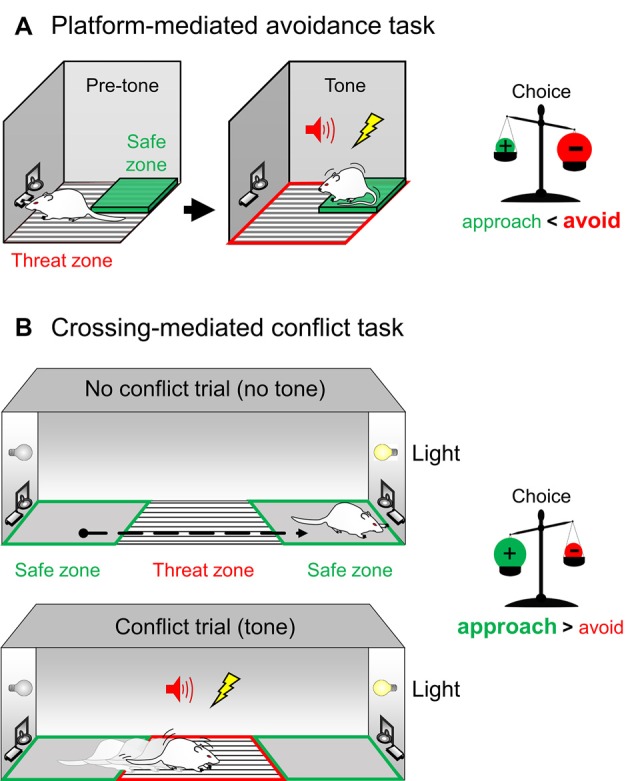
Novel behavioral assays to probe competing aversive and reward memories during a conflict. **(A)** *Left*. In the platform-mediated avoidance (PMA) task, rodents pressing a lever for reward are presented with a warning tone that predicts the onset of a foot-shock. Rodents then must step onto a safety platform opposite to the reward dispenser to avoid the punishment. PMA poses a conflict for rodents, where they must choose to seek safety during the warning signal at the expense of reward attainment during that period. *Right*. Thus, in PMA, during tone presentation the choice is balanced towards expressing the aversive negative memory (+) over the reward positive memory (−), leading the animal to avoid the grid rather than approach the reward (the avoidance drive is stronger than the approach drive). **(B)** *Left*. In the crossing-mediated conflict (CMC) task, a rodent trained to press a lever for reward is placed on one end of a straight alley and must cross a grid to obtain a light-signaled reward on the safe zone on the opposite side of the alley (no-conflict trials). However, the same animal also learns that a warning tone predicts a foot-shock in the threat zone (conflict trials). CMC poses a conflict for rodents, where they must discriminate between trials and choose to overcome fear to obtain a reward. *Right*. Thus, in CMC, during tone-signaled conflict trials, the choice is balanced towards expressing the reward positive memory (+) over the aversive negative memory (−), leading the animal to cross the grid to obtain the reward despite the threat (the approach drive is stronger than the avoidance drive).

One of our tasks requires rodents to choose whether to step into a safe platform to avoid the threat, while the other task requires rodents to choose whether to cross a threat zone to obtain a reward. Both tasks involve an approach-avoidance conflict and a cost-benefit decision guided by competing emotional memories. The platform-mediated avoidance (PMA) task serves a model of risky-reward seeking, in which animals foraging reward in the corner of a behavioral box must go onto a safety platform on the opposite corner to avoid a tone-signaled foot-shock (Bravo-Rivera et al., 2014, 2016; Figure 2A). The advantages and disadvantages of PMA compared to other avoidance tasks have been described elsewhere (Diehl et al., 2019). In the crossing-mediated conflict (CMC) task, an animal that is placed on one end of a straight alley must cross a grid to obtain a light-signaled reward on the safe zone on the opposite side of the alley. However, the animal also learns that a sound signals the presence of a foot-shock in the grid (threat zone). This task allows for discrimination of no-conflict trials (crossings when obtaining reward does not have a cost) and conflict trials (crossings that challenge the animal to take action to face the threat in order to obtain the reward; Hernandez-Jaramillo and Sotres-Bayon, 2018; Illescas-Huerta et al., 2018; Figure 2B). The CMC task is similar to the task employed by Olds (1958) in his classical self-stimulation studies, but this model of active suppression of fear remains largely unstudied and unmodeled. The key difference between PMA and CMC tasks is that in the former animals seek reward while avoiding threat and, in the latter, animals must actively overcome the threat to execute reward-seeking behavior. In PMA, the threat is a risk while in CMC, the threat is imminent and must be faced. Notably, both tasks allow identifying risk-prone or “courageous” and risk-taker or “cowardly” traits in rats. These features make these conflict tasks suitable to study brain circuit mechanisms that underlie the ability of animals to execute opposing behavioral responses guided by competing emotional memories.

## Maladaptive Emotional Decision-Making in Humans

Individuals weigh benefits of rewards against their associated costs to make more advantageous decisions (Hu, 2016). There are, however, crippling mental disorders that impair an individual’s ability to make appropriate decisions when presented with risky-reward opportunities. A major depressive disorder is characterized by a persistently depressed mood. Patients are unmotivated to seek rewards, which impairs the quality of life (American-Psychiatric-Association, 2013). Depressive patients tend to have exaggerated perceptions of punishment (Hevey et al., 2017), which leads to excessive avoidance and perpetuate their symptoms (Trew, 2011). Another mental disorder that impairs decisions regarding risky-reward situations is addiction. Addiction patients have exaggerated perceptions of reward and decreased sensitivity to punishment, which often results in neglecting the aversive consequences that addictions entail, such as health detriment or social problems (Myers et al., 2017). Characterizing motivation circuits that govern approach/avoidance conflict behavior in rodents is an important step to understand the underpinnings of these emotional disorders in humans.

## Author Contributions

CB-R and FS-B wrote the manuscript.

## Conflict of Interest

The authors declare that the research was conducted in the absence of any commercial or financial relationships that could be construed as a potential conflict of interest.
